# Perioperative mortality and 1-year neurodevelopmental outcome after cardiac surgery prior to 6 weeks of age, requiring perioperative extracorporeal membrane oxygenation in the first year of life

**DOI:** 10.3389/fcvm.2026.1828474

**Published:** 2026-06-26

**Authors:** Reto Engeli, Walter Knirsch, Verena Rathke, Michael von Rhein, Christelle L’Ebraly, Sebastian Grunt, Janet F. Kelly-Geyer

**Affiliations:** 1 Pediatric Cardiology, Pediatric Heart Center, Children’s Research Center, University Children’s Hospital, University of Zurich, Zurich, Switzerland; 2 Child Development Center, Children’s Research Center, University Children’s Hospital, University of Zurich, Zurich, Switzerland; 3 Pediatric Cardiology, Woman-Mother-Child Department, University Hospital Lausanne, Lausanne, Switzerland; 4 Pediatric Cardiology, Woman-Child-Adolescent Department, University Hospitals and Faculty of Medicine, University of Geneva, Geneva, Switzerland; 5 Pediatric Neurology, University Children’s Hospital, University of Bern, Bern, Switzerland; 6 Department of Pediatric Intensive Care and Neonatology, Children’s Research Center, University Children’s Hospital, University of Zurich, Zurich, Switzerland

**Keywords:** congenital heart disease, extracorporeal membrane oxygenation, mortality, neonatal cardiac surgery, neurodevelopmental outcome

## Abstract

**Objectives:**

Complex congenital heart disease (CHD), neonatal cardiac surgery, and perioperative extracorporeal membrane oxygenation (ECMO) are risk factors for increased perioperative mortality and morbidity, including impaired neurodevelopmental (ND) outcomes. We investigated perioperative mortality and the independent impact of ECMO on 1-year ND outcomes after cardiac surgery in infants younger than 6 weeks.

**Methods:**

Using data from the Swiss Outcome Registry for Children with severe CHD (Swiss ORCHID), we assessed the impact of perioperative ECMO on 1-year mortality and ND outcomes using the Bayley Scales of Infant Development, Third Edition (BSID III) and language composite scores (LCSs), cognitive composite scores (CCSs), and motor composite scores (MCSs) while controlling for known risk factors.

**Results:**

The 1-year mortality rate among 240 patients who underwent surgery between 1 January 2020 and 30 May 2024 was 5.8%. Perioperative ECMO was used in 34 patients (14.2%). Mortality among patients with perioperative ECMO (ECMO group) was higher (29.4% in the ECMO group vs. 1.9% in the non-ECMO group, *p* < 0.001) and was more frequently associated with univentricular CHD (*p* = 0.005). The mean BSID III CCS (*β*=−13.942, SE = 3.736, *p* = <0.001) and MCS (*β* = −15.913, SE = 4.325, *p*=<0.001) were significantly lower in the ECMO group compared to the non-ECMO group in simple linear regression analyses. Multiple linear regression showed a negative impact of ECMO on CCS (*p* < 0.05); MCS was influenced by longer hospital stay (*p* < 0.01), and lower socioeconomic status (*p* < 0.05).

**Conclusions:**

Perioperative ECMO might be associated with higher perioperative mortality and morbidity, including lower BSID III ND scores in infants with CHD.

**Clinical trial registration:**

clinicaltrials.gov, NCT05996211.

## Introduction

Extracorporeal membrane oxygenation (ECMO) is widely used in pediatric patients with congenital heart disease (CHD) for perioperative cardiorespiratory support (1–4). While ECMO is potentially lifesaving, its use and duration are limited by a high rate of neurological complications, including impaired neurodevelopmental (ND) outcomes (3–7). Therefore, perioperative ECMO is considered a risk factor for impaired ND outcomes in infants requiring surgery for severe CHD (8). In addition to ECMO, other perioperative risk factors have recently been categorized as either modifiable or non-modifiable (9). However, the impact of perioperative ECMO on the mid-term ND outcomes of these infants remains unclear and warrants further investigation (10, 11).

## Methods

### Data source and patient population

The cantonal ethics committee gave ethical approval for this study (BASEC-number: 2024-00130) after the ORCHID registry had been approved by the responsible cantonal ethics committees (Req-2019-00089) and was registered at clinicaltrials.gov. This cohort study was reported in line with the Strengthening the Reporting of Observational Studies in Epidemiology (STROBE) statement (Supplementary Data Sheet 2) (12).

The Swiss ND Outcome Registry for Children with Severe CHD (Swiss ORCHID) registry collects perioperative variables and ND outcome data at follow-up and was used for this analysis (13). The Swiss ORCHID is a nationwide clinical patient registry including neonates and young infants with severe CHD undergoing cardiac surgery or catheter-based procedures within the first 6 weeks of life. It has prospectively collected data from pediatric cardiac and ND follow-up centers all over Switzerland since 2019. For this analysis, we included all consecutive patients recruited after birth whose parents or guardians provided written informed consent. First, we analyzed mortality in the first year of life (defined as death before the 1-year ND outcome assessment) among patients in the Swiss ORCHID cohort and the impact of perioperative ECMO. Second, we analyzed the ND outcome at 1 year of age using the Bayley Scales of Infant and Toddler Development, 3rd ed. (BSID III), comparing patients with and without perioperative ECMO. For this analysis, we excluded patients with a known genetic comorbidity and incomplete or missing ND outcome data (BSID III). We hypothesized that there would be an adverse impact of perioperative ECMO on both mortality and morbidity.

### Procedures

Procedure I was defined as the first surgical or catheter-based procedure performed after birth at less than 6 weeks of age. Procedure II was defined as a secondary surgical procedure during the first year of life, either in patients undergoing staged single ventricle palliation with a bidirectional cavopulmonary anastomosis (Glenn) for single ventricle CHD, or secondary cardiac surgery in staged complex biventricular repair, or those who required secondary cardiac procedures due to residual cardiac lesions.

The complexity of cardiac surgery was rated from one to six using the Risk Adjustment for Congenital Heart Surgery (RACHS-1) score (14). RACHS-1 scores from one to three were defined as low, and scores from four to six as high (15). The congenital heart defects were classified using the four distinct primary cardiac diagnosis classes described by Clancy et al. (16): class I, biventricular heart without arch obstruction; class II, biventricular heart with arch obstruction; class III, single-ventricle heart without arch obstruction; class IV, single-ventricle heart with arch obstruction.

### ECMO

ECMO was used at different times, which we categorized as follows: (1) preoperatively, (2) postoperatively starting in the operating room (primary ECMO), (3) postoperatively on the intensitve care unit (ICU) due to a therapy-refractory cardiorespiratory deterioration (secondary ECMO), or (4) during resuscitation as extracorporeal resuscitation (ECPR). ECPR was defined as the use of ECMO during cardiopulmonary resuscitation or within 20 min of the return of spontaneous circulation. Furthermore, we analyzed the cannulation technique (central vs. peripheral), the type of ECMO used (venoarterial [VA] vs. venovenous [VV], and the total duration of the ECMO runs.

### Complications

Perioperative complications were defined as low cardiac output syndrome, respiratory complications (e.g., pleural effusions requiring drainage, pneumothorax, chylothorax, hemothorax, and diaphragmatic paralysis); wound infection, necrotizing enterocolitis, neurological complications (e.g., cerebral stroke, clinical or subclinical cerebral seizures, and pathologic cerebral imaging findings) and multiorgan failure defined as failure of more than one organ. We calculated the total length of hospital stay (LoHS) as the sum of the hospital stays during procedures I and II.

### ND outcome at 1 year of age

ND outcome was assessed at 1 year of age in follow-up centers by trained developmental pediatricians using the (17) language (LCS), cognitive (CCS), and motor (MCS) composite scores of the BSID III at routine follow-up visits. The BSID III has a mean of 100 points with a standard deviation (SD) of 15 points (18). Socioeconomic status (SES) was rated on a 6-point rating scale based on paternal occupation and maternal education (19).

### Statistical analysis

Statistical analysis was performed using R software (R: A Language and Environment for Statistical Computing, R Core Team, R Foundation for Statistical Computing, Vienna, Austria, 2023, https://www.R-project.org) along with the lm() function from the “stats” package (version 4.3.2). Further statistical details are provided in Supplementary Material A.

## Results

### Mortality

**Figure 1:**
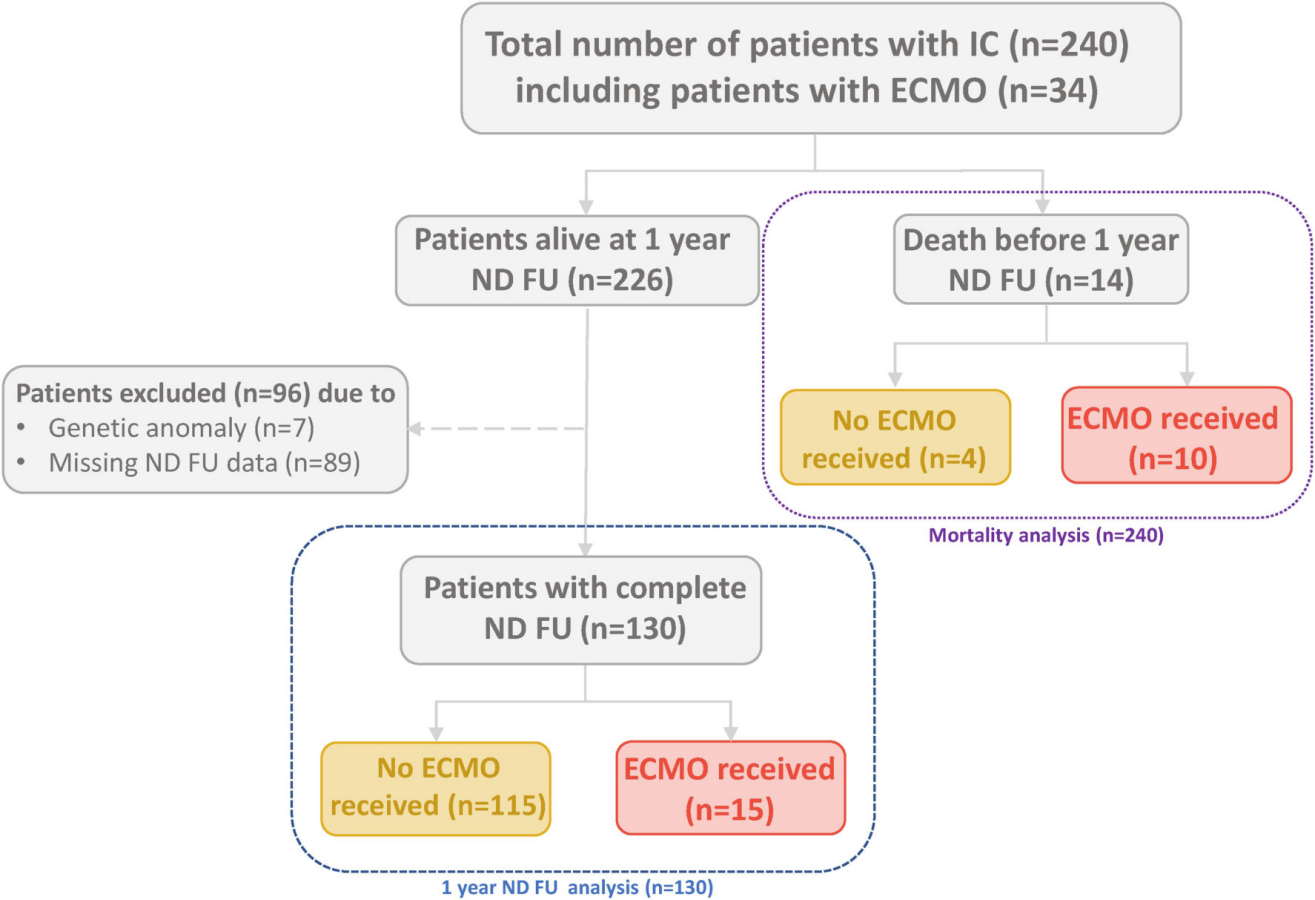
Patient flow chart for mortality analysis (*n* = 240) and 1-year neurodevelopmental outcome follow-up analysis (*n* = 130). ECMO, extracorporeal membrane oxygenation; FU, follow-up; IC, informed consent; ND, neurodevelopmental.

We report on the total number of patients (*n* = 240) included between 1 January 2020 and 30 May 2024 (Figure 1). The mortality rate was 6% (14 of 240 patients) within the first year of life. Perioperative ECMO was performed in 34 of 240 patients (14%). The 1-year mortality among patients with perioperative ECMO (ECMO group) was significantly higher than that of those without ECMO [29% (10 of 34) vs. 2% (4 of 206), *p* < 0.001].

Of the 240 infants considered for the mortality analysis, 184 had biventricular cardiac anatomy, and 56 had univentricular anatomy. The 1-year mortality of univentricular CHD patients was 14% (8 of 56), which was significantly higher than that of biventricular patients [3% (6 of 184); *p* = 0.005], independent of ECMO. The 1-year mortality was higher after perioperative ECMO in patients with univentricular CHD [43% (6 of 14) vs. 5% (2 of 42) in patients with univentricular CHD without ECMO, *p* = 0.002]. In comparison, mortality among patients with biventricular CHD and ECMO was 20% (4 of 20) vs. 1% (2 of 164) among those without ECMO (*p* = 0.001).

VA-ECMO was used in 91% (31 of 34) of patients with a mortality rate of 29% (9 of 31), whereas VV-ECMO was used in 9% (3 of 34) of patients with no mortality. ECPR was performed in 15% (5 of 34) of patients with ECMO, with a mortality rate of 40% (2 of 5). Further details are provided in Supplementary Table S1.

### Procedures

We analyzed the impact of ECMO on the ND outcome in 130 patients with complete 1-year ND outcome data (Figure 1). The patient characteristics are detailed in Table 1. Twelve percent (15 of 130) of this cohort received perioperative ECMO. Eighty-six percent (112 of 130) of patients underwent cardiopulmonary bypass (CPB) surgery for procedure I at a median age of 7 days [interquartile range (IQR): 5 to 14 days] (see Supplementary Table S1). Five infants had an interventional cardiac catheterization at procedure I, one patient had a combined catheter-based and surgical (hybrid) procedure, and the remaining patients received cardiac non-bypass surgery as part of univentricular palliation, all without perioperative ECMO. Ninety-two percent of patients who received ECMO during procedure I (11 of 12) had a high RACHS score (4–6), compared to 41% (48 of 118) of those who did not receive ECMO during procedure I (*p* = 0.0014). The median (IQR) CPB time at procedure I was 221 min (166–282), with a significantly longer CPB duration in the ECMO group than in the non-ECMO group [328 min (238–362) vs. 216 min (160–268), *p* = 0.0004]. The median cross-clamp time was 130 min (80–164). CPB surgery with anterograde cerebral perfusion was performed in 43% (56 of 130) of patients.

**Table 1:** Patient characteristics.

Parameter^a^	Patients, total (*n* = 130)	Non-ECMO (*n* = 115)	ECMO (*n* = 15)	*p*
Patient characteristics				
Sex (male)	83 (64%)	74 (64%)	9 (60%)	0.779
GA (weeks)	38.93 (38.18–40.00)	38.86 (38.29–40)	39.43 (38.14–39.93)	0.907
BW (kg)	3.30 (2.96–3.60)	3.31 (2.96–3.63)	3.2 (3.08–3.50)	0.613
SES score, total	4 (3.00–6.00)	5 (3.00–6.00)	4 (3.75–4.25)	0.301
Type of CHD				0.207
Biventricular	97 (75%)	88 (77%)	9 (60%)	
Univentricular	33 (25%)	27 (24%)	6 (40%)	
Primary cardiac diagnosis				0.274
Clancy Class 1	74 (57%)	68 (59%)	6 (40%)	
Clancy Class 2	23 (18%)	20 (17%)	3 (20%)	
Clancy Class 3	16 (12%)	14 (12%)	2 (13%)	
Clancy Class 4	17 (13%)	13 (11%)	4 (27%)	
Complications				
Complications, total	81 (62%)	67 (58%)	14 (93%)	**0.009**
Neurological complications	49 (38%)	37 (32%)	12 (80%)	**<0.001**
Pathological cerebral MRI	36 (28%)	26 (23%)	10 (67%)	
Pathological cerebral US	17 (13%)	16 (14%)	1 (7%)	
Clinical seizure	4 (3%)	2 (2%)	2 (13%)	
Subclinical seizure	1 (1%)	1 (1%)	NA	
Cerebral stroke	2 (2%)	1 (1%)	1 (7%)	
Resuscitation, total	18 (14%)	12 (10%)	6 (40%)	**0.007**
LOHS, total (days)	45 (27–67)	42 (27–60)	103 (83–148)	**<0.001**
ICU time, total (days])	12 (7–25)	11 (7–19)	49 (33–80)	**<0.001**

Data are reported as median (IQR) and absolute numbers (percentages).

BW, birth weight; CHD, congenital heart disease; ECMO, extracorporeal membrane oxygenation; GA, gestational age; ICU, intensive care unit; LOHS, length of hospital stay; MRI, magnetic resonance imaging; SES, socioeconomic status; US, ultrasound.

a Data given only for patients that had data for the respective parameter.

Bold values are determined as p <0.05.

Forty-six patients underwent procedure II surgery with CPB at an age of 163 days (128–217). Seventeen percent of non-ECMO patients (7 of 42) and none of the ECMO procedure II patients had a high RACHS score. Procedure II surgery was performed with a median CPB time of 156 min (118–243) and a cross-clamp time of 67 min (40–136).

Twelve percent (15 of 130) of the patient cohort had a cardiac reoperation during their hospital stay: 10% (12 of 118) of the non-ECMO patients and 25% (3 of 12) of the patients with ECMO. Eleven percent (5 of 46) of patients had cardiac reoperation during their procedure II hospital stay; 10% were non-ECMO (4 of 42), and 25% were ECMO patients (1 of 4).

Twelve patients in the ECMO group received ECMO during procedure I, with an ECMO duration of 87 h (72–123). All these patients were centrally cannulated, and all but one patient had a single run of VA-ECMO. One infant additionally received VV–ECMO. Half of the ECMO runs (6 of 12) were started electively in the operating room (primary ECMO), 42% (5 of 12) after surgery in the ICU (secondary ECMO), and 8% (1 of 12) preoperatively.

Four patients received ECMO during procedure II. The ECMO duration during procedure II was 96 h (78–130). All patients were centrally cannulated, three of four patients received VA-ECMO, and one patient received VV. ECMO was initiated as either primary (*n* = 2) or secondary (*n* = 2). One patient received ECMO during both procedures I and II. ECPR was performed in 17% (2 of 12) of ECMO patients for procedure I and in 25% (1 of 4) in procedure II.

### Complications

Sixty-two percent of patients (81 out of 130) experienced a complication either during procedure I and/or procedure II (Table 1). Seventy-seven of 130 (59%) patients had a complication after procedure I, and 20 of 46 (44%) patients after procedure II. In the ECMO group, neurological complications were significantly higher [ECMO group 80% (12 of 15) vs. non-ECMO group 32% (37 of 115), *p* < 0.001], as was the resuscitation rate [resuscitation in ECMO group 40% (6 of 15) vs. non ECMO group 10% (12 of 115), *p* = 0.007], the total ICU length of stay [ECMO group 49 days (33–80) vs. non-ECMO group 11 (7–19) *p* < 0.001], and total LoHS [ECMO group 103 days (83–148) vs. non-ECMO group 42 days (27–60), *p* < 0.001] (Table 1).

### ND outcome at 1 year of age

**Figure 2:**
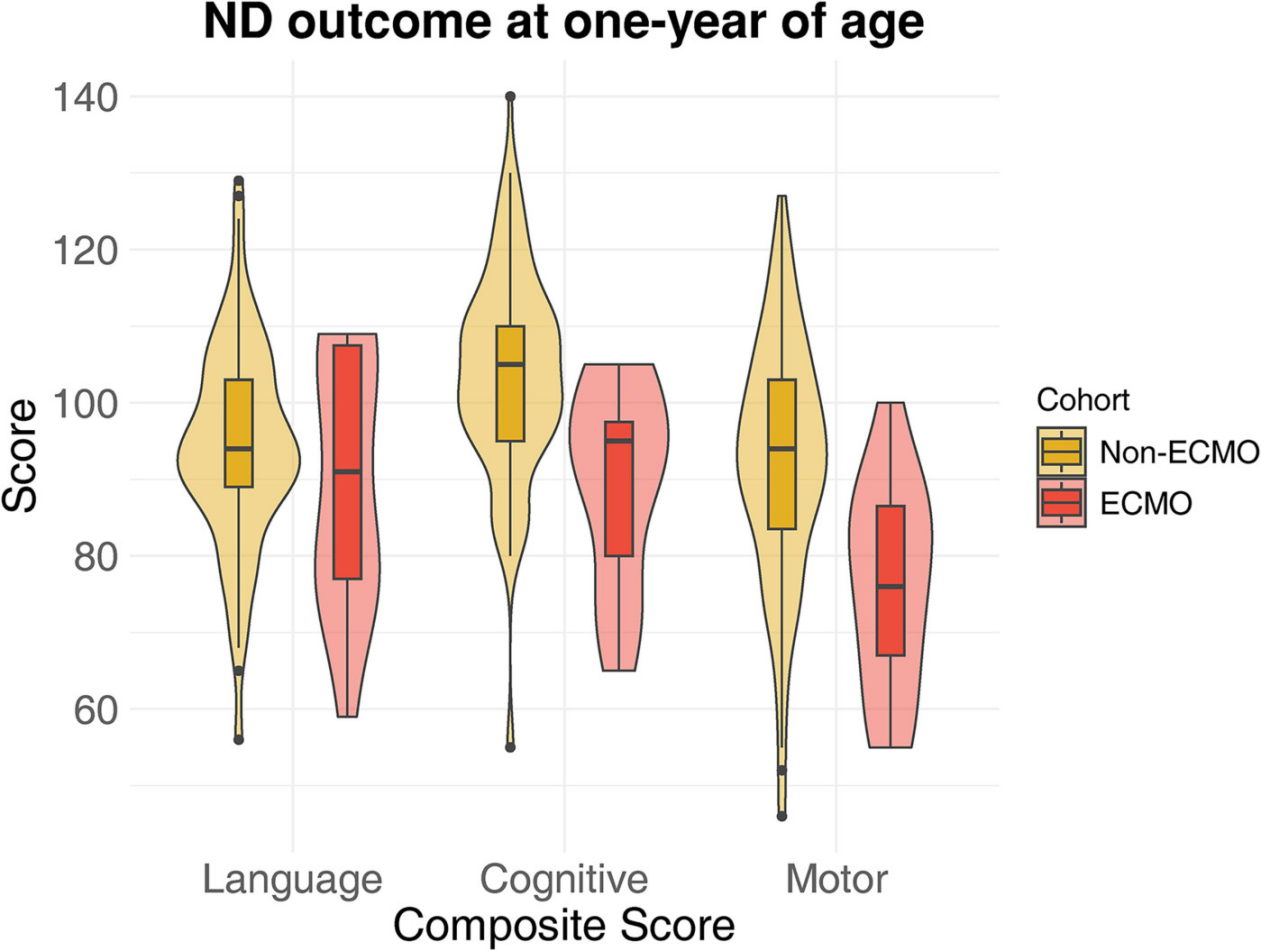
Comparison of neurodevelopmental outcomes at 1-year of age in infants undergoing cardiac surgery in the first 6 weeks of life with or without perioperative ECMO in the first year of life. ECMO, extracorporeal membrane oxygenation; LCS, language composite score (BSID III); CCS, cognitive composite score (BSID III); MCS, motoric composite score (BSID III).

Median BSID III composite scores across all three domains were lower in the ECMO group than in the non-ECMO group (Figure 2).

Simple linear regression demonstrated a negative impact of ECMO on the CCS (*β* = −13.942, SE = 3.736, *p* < 0.001) and the MCS (*β* = −15.913, SE = 4.325, *p* < 0.001), respectively (Table 2). No evidence was found for an influence of ECMO on the LCS (*β* = −4.649, SE = 3.762, *p* = 0.219) (Table 2).

**Table 2:** Neurodevelopmental outcomes at 1 year of age.

Parameter^a^	Patients, total (*n* = 130)	Non-ECMO (*n* = 115)	ECMO (*n* = 15)	*p*
Age at ND outcome assessment (months)	12 (11–13)	12 (11–12)	11 (11–13)	0.844
Language composite score	94 (86–103)	94 (89–103)	91 (77–108)	0.219
BSID III EC scaled score	9 (8–10)	9 (8–10)	9 (6–10)	0.274
BSID III RC scaled score	9 (7–11)	9 (7–11)	8 (6–12)	0.411
Cognitive composite score	100 (95–110)	105 (95–110)	95 (80–98)	**<0.001**
BSID III cognitive scaled score	10 (9–12)	11 (9–12)	9 (6–10)	**<0.001**
Motor cognitive score	91 (80–100)	94 (84–103)	76 (67–86)	**<0.001**
BSID III fine motoric scaled score	10 (9–11)	10 (9–12)	9 (7–10)	**0.038**
BSID III gross motor scaled score	7 (4–9)	8 (5–9)	3 (1–6)	**<0.001**
Somatic growth at ND assessment				
Body length (z-score)	−0.11 (−1.07–0.63)	−0.08 (−0.83–0.67)	−1.06 (−1.91–−0.09)	**0.023**
Body weight (z-score)	0.00 (−0.9–0.7)	0.05 (−0.7–0.78)	−0.72 (−1.76–−0.35)	**0.006**
Head circumference (z-score)	−0.49 (−1.34–0.52)	−0.17 (−1.25–0.58)	−1.67 (−3.2–−0.87)	**<0.001**

Data are reported as median (IQR) and absolute numbers.

Age at ND outcome assessment was corrected for prematurity.

ECMO, extracorporeal membrane oxygenation; EC, expressive communication; RC, receptive communication.

a Data given only for patients that had data for the respective parameter.

Bold values are determined as p <0.05.

Multivariate analysis showed a significant negative effect of ECMO exposure on the CCS (*β* = −8.836, SE = 4.431, *p* = 0.048). Higher BW (*β* = 6.442, SE = 2.510, *p* = 0.012) positively influenced the CCS. Longer LoHS (*β* = −0.104, SE = 0.033, *p* = 0.002) and a lower SES (*β* = −1.333, SE = 0.601, *p* = 0.029 negatively affected the MCS. No predictors significantly influenced the LCS (Table 2).

## Discussion

We analyzed the impact of perioperative ECMO on mortality and 1-year ND outcomes in neonates and young infants undergoing cardiac surgery for severe types of CHD.

We determined an overall 1-year mortality of 6% for this patient cohort, which is less than that reported in a large recent European study (9.1% in-hospital mortality) (20). As previously described (4, 21), 1-year mortality was significantly higher in patients with univentricular CHD compared with those with biventricular CHD (*p* = 0.005), and in those who required perioperative ECMO compared with those who did not (29% vs. 2%, *p* < 0.001). The use of perioperative ECMO was high (14%, 34 of 240), ECPR was necessary in 15% of cases, and was associated with the highest mortality (40%).

Worldwide, 2% to 5% of children undergoing cardiac surgery receive perioperative ECMO (5, 20, 22–25). Survival to hospital discharge in children with CHD requiring perioperative ECMO ranges between 40% and 49%, depending on the complexity of the patient cohort (5, 22–25). The high number of patients with perioperative ECMO in our patient cohort may be explained by the severity of the CHD in this predominantly neonatal patient cohort and the high proportion of infants with univentricular anatomy (25%). Infants with single ventricle physiology are known to require perioperative ECMO more frequently and have higher mortality rates (3, 21, 26).

Infants with severe CHD have many risk factors for perioperative mortality and long-term morbidity, including adverse ND outcomes (27). These include non-modifiable risk factors such as genetic syndromes and the effects of CHD itself, in addition to both cardiac and non-cardiac complications, such as requiring resuscitation or perioperative ECMO-related neurological complications (9, 27, 28). Our ECMO patient cohort had a higher rate of additional risk factors for impaired ND outcome compared with the non-ECMO group. Of note, significant postoperative *residual cardiac lesions* requiring reoperation were present in 50% of patients undergoing perioperative ECMO during the hospital stay for procedure II, compared to 11% of patients with non-ECMO. During the hospital stay for procedure I, 25% of patients in the ECMO group required cardiac reoperation, compared with 10% of the non-ECMO group. The higher incidence of postoperative ECMO in patients with residual cardiac lesions has been described. In addition, these infants require an additional CPB procedure which represents an additional risk factor for impaired ND outcome (29, 30).

Furthermore, infants requiring *resuscitation* typically experience a period of decreased cerebral perfusion and hypoxia, increasing the risk of brain injury and ND impairment (5, 31). It is likely that not only the survival, but also the ND outcome of infants after ECPR is unfavorable compared with infants receiving ECMO not associated with resuscitation (29, 32, 33). Forty percent of the infants in our ECMO group were resuscitated compared to only 10% of the non-ECMO group.

These *additional risk factors* for impaired ND outcome in the ECMO group, together with the significantly increased LoHS in this patient cohort, likely indicate that this cohort had a generally higher severity of illness than the non-ECMO cohort. LoHS has been clearly shown to be associated with worse ND outcome (27, 34, 35). All these risk factors make it more challenging to determine the additional effect of ECMO on ND outcomes. However, ECMO itself is associated with a high risk of neurological complications, especially seizures, ischemic stroke, and intracerebral bleeding. There is clear evidence that neurological complications on ECMO are associated with worse ND outcomes (6, 36, 37).

Eighty percent of the infants in our ECMO group had neurological complications (NC), defined as a clinical or sub-clinical seizure, stroke, or abnormal cerebral imaging, compared with 32% in the non-ECMO group, which is high compared to the literature (5, 45). Our definition of NC also included mild cerebral findings (e.g., microbleeds) detected on cerebral MRI, for which the impact on ND outcome is likely to be more limited (46).

The prevalence of *ND impairment* following neonatal ECMO is between 10% and 60% (2, 5, 38), while the survival rate has been reported as 30.9% at the age of 4.4 years (5). Of the survivors, 49.9% had ND impairments, with 24.2% having severe cognitive problems, which is much higher than described in our study (5). The differing impact of ECMO on 1-year ND outcome has been described for cognitive and motoric outcomes. Sadhwani et al. also found a trend toward lower CCS in the ECMO group (1). Of note, they found the largest difference in the gross motor domain (1). The infants exposed to ECMO in our cohort had an MCS of 76 (67–86), indicating a risk for mild ND impairement. Similar to Quadir et al., we observed a trend toward major deficits in the gross motoric domain in the ECMO group (18). Furthermore, in our cohort, motor deficits were associated with longer total ICU stay and total LoHS, but not with ECMO status (Table 1). This may be attributed to the small sample size or to the MCS model’s controlling for LoHS as a partial surrogate for severity of illness. LoHS may act as a confounder, reflecting differences in illness severity between the ECMO and non-ECMO cohorts (Table 3). Including LoHS as a parameter in the multivariable analysis may potentially reduce this bias. The timing and criteria for perioperative ECMO in infants with severe CHD were not standardized from center to center (4).

**Table 3:** Simple and multiple linear regression model of ND outcome at 1 year of age.

	CCS					MCS					LCS				
	*β*	SE	Lower CI (2.5%)	Upper CI (97.5%)	*p*	*β*	SE	Lower CI (2.5%)	Upper CI (97.5%)	*p*	*β*	SE	Lower CI (2.5%)	Upper CI (97.5%)	*p*
Simple linear regression															
Intercept	103.609	1.269	101.098	106.119	**<0.001**	92.113	1.469	89.206	95.02	**<0.001**	94.183	1.278	91.654	96.711	**<0.001**
Cohort (ECMO vs. non-ECMO)	−13.942	3.736	−21.333	−6.551	**<0.001**	−15.913	4.325	−24.471	−7.355	**<0.001**	−4.649	3.762	−12.094	2.795	0.219
Multiple linear regression															
Intercept	88.426	9.195	70.218	106.635	**<0.001**	98.008	10.278	77.654	118.361	**<0.001**	88.629	9.568	69.68	107.579	**<0.001**
Total LoHS (days)	−0.028	0.030	−0.086	0.031	0.351	−0.104	0.033	−0.169	−0.038	**0.002**	−0.042	0.031	−0.103	0.019	0.173
Resuscitation (yes vs. no)	−0.778	3.592	−7.890	6.334	0.829	−6.352	4.016	−14.305	1.601	0.116	−2.099	3.725	−9.475	5.276	0.574
NC (yes vs. no)	−3.634	2.600	−8.782	1.514	0.165	−2.147	2.904	−7.897	3.603	0.461	1.231	2.697	−4.108	6.571	0.649
Type of CHD (univentricular vs. biventricular)	−4.046	3.025	−10.036	1.944	0.184	−0.807	3.381	−7.502	5.888	0.812	−1.893	3.137	−8.104	4.318	0.547
SES score total	−0.457	0.539	−1.526	0.613	0.399	−1.333	0.601	−2.525	−0.14	**0.029**	−0.342	0.575	−1.485	0.801	0.554
BW (kg)	6.442	2.510	1.471	11.412	**0.012**	1.763	2.804	−3.789	7.315	0.531	3.692	2.603	−1.462	8.847	0.159
Sex (male vs. female)	−0.117	2.465	−4.997	4.764	0.962	2.496	2.755	−2.959	7.951	0.367	−3.845	2.556	−8.906	1.216	0.135
Cohort (ECMO vs. non-ECMO)	−8.836	4.431	−17.61	−0.061	**0.048**	−4.741	4.953	−14.549	5.067	0.340	−0.830	4.596	−9.93	8.269	0.857

*β*, estimated beta; BW, birth weight; CCS, cognitive composite score (BSID III); CHD, congenital heart disease; CI, confidence interval; ECMO, extracorporeal membrane oxygenation; LCS, language composite score; LoHS, length of hospital stay; MCS, motoric composite score (BSID III); NC, neurological complication; SE, standard error; SES, socioeconomic status.

Bold values are determined as p <0.05.

When assessing the impact of ECMO exposure on the 1-year ND outcome as a single variable, we found a negative effect on the BSID-III MCS and CCS. The negative effect of ECMO persisted in the CCS after controlling for other factors influencing ND outcomes. The LCS was not affected by ECMO exposure (Figure 1). While most studies show a negative impact of ECMO on the cognitive outcome in around 50% of patients, motor and language development seem less affected (1, 39).

Nevertheless, we found strong evidence in the multivariable analysis that longer total LoHS negatively affects the MCS. The longer hospital stay might have limited the child’s mobility, thereby inhibiting gross motor learning. Furthermore, we found evidence of a negative association between lower SES and MCS. A lower SES (40) and longer total LoHS have both been consistently determined as risk factors for impaired ND outcomes in CHD patients (34, 35, 41, 42). Higher educational status might enable parents to better understand the medical and ND needs of their child, and they therefore might initiate therapy earlier, potentially improving ND outcomes (Table 1).

The infants in the ECMO group had lower somatic growth in weight, length, and head circumference at the time of follow-up, with a mean Z-score of −2 for head circumference compared to the non-ECMO group, which had a Z-score of 0 (Table 2). This could provide further evidence that these children have been generally sicker than the patients in the non-ECMO cohort without catch-up growth before the assessment. Reduced growth in infants with CDH has been associated with worse ND outcomes, especially in the cognitive and motor domains (27, 43).

### Limitations

This study has several limitations. First, there was a small absolute number of patients with perioperative ECMO (*n* = 15) compared with the large number of patients with heterogeneous types of CHD in the non ECMO group (*n* = 115). Second, treatment in four different pediatric heart centers makes it difficult to assume absolute standardization of surgical and perioperative management, including the indication for and timing of perioperative ECMO. Third, the statistical power was limited due to unbalanced group sizes and the presence of multiple risk factors influencing perioperative mortality and morbidity, as well as the ND outcome. Fourth, the reported survival rates in our overall cohort (94.2%) and in the ECMO cohort (71%) may be overestimated because the ORCHID registry policy requires written informed consent. This excluded patients without informed consent due to early postnatal death or the difficulty of obtaining consent during critical illness. Fifth, the ND outcome has only been analyzed at one year of age, which is less sensitive than analyses of older children, particularly in the cognitive and language domains (42, 44).

## Conclusions

In infants with severe CHD undergoing early cardiac surgery, perioperative mortality is higher, and ND outcomes in the cognitive and motor domains are worse after ECMO. After controlling for known risk factors, some evidence was found of worse ND outcomes in the cognitive domain of patients with ECMO. Larger studies are required to elucidate the contribution of ECMO and other potential risk factors to ND outcomes in patients with CHD.

ECMO remains a life-saving treatment option for infants with CHD and therapy-refractory cardiorespiratory failure. However, due to the increased risk of impaired ND outcome, the threshold for instituting ECMO should remain high.

## Data Availability

The dataset presented in this paper is not readily available due to ethical regulations. Requests to access the datasets should be directed to the corresponding author/s.
