# Supplementary material for: Perioperative mortality and 1-year neurodevelopmental outcome after cardiac surgery prior to 6 weeks of age, requiring perioperative extracorporeal membrane oxygenation in the first year of life
Source: Front Cardiovasc Med. 2026 Jun 26;13:1828474. doi: 10.3389/fcvm.2026.1828474 (PMC13350321; doi:10.3389/fcvm.2026.1828474)
Supplement: Supplementary file 1 [file Table1.docx]

**Supplemental Table 1.** Surgical parameters of procedure I and II for infants with ND follow up at 1 year of age.

|  | **Procedure I** | | |  | **Procedure II** | | |  |
| --- | --- | --- | --- | --- | --- | --- | --- | --- |
| **Parameter**^#^ | **Patients, total  (n = 130)** | **Non-ECMO  (n = 118)** | **ECMO  (n = 12)** | **P** | **Patients, total (n= 46)** | **Non-ECMO  (n = 42)** | **ECMO  (n = 4)** | **P** |
| Age at surgery [days] | 7 (5 - 14) | 7 (5 - 14) | 8 (7 - 14) | 0.364 | 163 (128 - 217) | 165 (130 - 217) | 88 (76 - 130) | 0.067 |
| Type of surgery (RACHS) |  |  |  | **< 0.001** |  |  |  | 0.551 |
| - I | 0 (0%) | 0 (0%) | 0 (0%) |  | 0 (0%) | 0 (0%) | 0 (0%) |  |
| - II | 15 (12%) | 15 (13%) | 0 (0%) |  | 22 (48%) | 21 (50%) | 1 (25%) |  |
| - III | 51 (39%) | 50 (42%) | 1 (8%) |  | 17 (37%) | 14 (33%) | 3 (75%) |  |
| - IV | 43 (33%) | 38 (32%) | 5 (42%) |  | 3 (7%) | 3 (7%) | 0 (0%) |  |
| - V | 1 (1%) | 0 (0%) | 1 (8%) |  | 0 (0%) | 0 (0%) | 0 (0%) |  |
| - VI | 15 (12%) | 10 (8%) | 5 (42%) |  | 4 (9%) | 4 (10%) | 0 (0%) |  |
| - No score (PDA stent only) | 5 (4%) | 5 (4%) | 0 (0%) |  | NA | NA | NA |  |
| CPB surgery | 112 (86%) | 100 (85%) | 12 (100%) | 0.781 | 46 (100%) | 42 (100%) | 4 (100%) | NA‡ |
| CPB time [min] | 221 (166 - 282) | 216 (160 - 268) | 328 (238 - 362) | **< 0.001** | 156 (118 - 243) | 146 (116 - 242) | 214 (194 - 258) | 0.133 |
| Anterograde cerebral perfusion | 56 (43%) | 50 (42%) | 6 (50%) | 0.761 | 10 (22%) | 9 (21%) | 1 (25%) | 1.000 |
| Anterograde cerebral perfusion duration [min] | 31 (24 - 63) | 30 (25 - 62) | 50 (22 - 94) | 0.747 | 43 (32 - 82) | 43 (32 - 82) | NA | NA‡ |
| Cross clamp time [min] | 130 (80 - 164) | 131 (76 - 160) | 102 (91 - 209) | 0.222 | 67 (40 - 136) | 66 (39 - 133) | 120 (91 - 132) | 0.474 |
| Lowest temperature during CPB [degrees Celsius] | 30 (27 - 32) | 30 (28 - 32) | 26 (24 - 27) | **0.002** | 33 (30 - 36) | 34 (31 - 36) | 25 (22 - 28) | NA‡ |
| LOHS, total [days] | 37 (24 - 56) | 35 (24 - 51) | 99 (70 - 116) | **< 0.001** | 21 (16 - 31) | 20 (15 - 30) | 101 (76 - 126) | **0.005** |
| LOS, ICU [days] | 9 (6 - 20) | 8 (6 - 16) | 41 (34 - 62) | **< 0.001** | 7 (5 - 13) | 7 (5 - 10) | 56 (42 - 69) | **0.002** |
| Total ECMO duration [hours] | NA | NA | 87 (72 - 123) |  | NA | NA | 96 (78 - 130) |  |
| Central cannulation | NA | NA | 12 (100%) |  | NA | NA | 4 (100%) |  |
| Venovenous ECMO | NA | NA | 1 (8%) |  | NA | NA | 1 (25%) |  |
| Venoarterial ECMO | NA | NA | 12 (100%) |  | NA | NA | 3 (75%) |  |
| ECMO pre OP | NA | NA | 1 (8%) |  | NA | NA | 0 (0%) |  |
| ECMO post OP primary (elective) | NA | NA | 6 (50%) |  | NA | NA | 2 (50%) |  |
| ECMO post OP secondary (ICU) | NA | NA | 5 (42%) |  | NA | NA | 2 (50%) |  |
| - ECPR | NA | NA | 2 (40%) |  | NA | NA | 1 (50%) |  |

^#^ *data given only for patients that had data for the respective parameter.* ^‡^ *less than 3 entries per group, p value cannot be calculated. Data are reported as median (IQR) and absolute numbers (percentages)*

This table summarizes all patients survived until 1 year ND follow up and splits the patients into procedure I and II. One patient has received ECMO in procedure I and II. As percentages are rounded to full numbers percentages might not add up to 100% for all parameters.

P, p-value; RACHS, Risk adjustment for congenital heart surgery; ECPR, Extracorporeal Cardiopulmonary Resuscitation; OP, operation; LOHS, length of hospital stay; LOS ICU, length of intensive care unit stay
