# Supplementary material for: Perioperative mortality and 1-year neurodevelopmental outcome after cardiac surgery prior to 6 weeks of age, requiring perioperative extracorporeal membrane oxygenation in the first year of life
Source: Front Cardiovasc Med. 2026 Jun 26;13:1828474. doi: 10.3389/fcvm.2026.1828474 (PMC13350321; doi:10.3389/fcvm.2026.1828474)
Supplement: Supplementary file 2 [file Datasheet1.pdf]

Supplement A.

In the simple linear regression model, LCS, CCS and MCS, respectively were used as the dependent variable, whereas ECMO exposure was chosen as the sole independent variable.

Multiple linear regression was performed with the function lm() of the stats package [stats] (version 4.3.2) and the pool function of the mice package [MICE] (version 3.16.0) [21,22]. The following independent variables were used to predict the respective BSID III composite scores: Total LOHS in days [10], resuscitation events [23], neurological complications [24], type of CHD at stage one [23], total SES score [10], birth weight (BW) [25], sex [26] and ECMO [23]. Missing numerical data was imputed with predictive mean matching and binary data with polytomous logistic regression, using the function mice() [MICE] [22,27]. Univariate analysis of numerical data between the two cohorts was conducted using an unpaired Student's t-test for normally distributed data with equal variances and Welch's t-test for normally distributed data without equal variances using t.test()[stats] [21]. Non-normally distributed numerical data was analyzed using two-sample Wilcoxon test wilcox.test() [stats] [21]. Categorical data was analyzed using Fisher’s exact test fisher.test() [stats] [21].

The significance level chosen was 0.05. Results of statistical analyses are reported as estimated betas (β), their standard error (SE) and confidence interval (CI) as well as the associated p. Data processing and visualization was done using the tidyverse package [tidyverse] (version 2.0.0) [28] in R.
