# Supplementary material for: Perioperative mortality and 1-year neurodevelopmental outcome after cardiac surgery prior to 6 weeks of age, requiring perioperative extracorporeal membrane oxygenation in the first year of life
Source: Front Cardiovasc Med. 2026 Jun 26;13:1828474. doi: 10.3389/fcvm.2026.1828474 (PMC13350321; doi:10.3389/fcvm.2026.1828474)
Supplement: Supplementary file 4 [file Datasheet3.pdf]

Collaborators (should be PubMed indexed on behalf of the Swiss ORCHID group [7])

Swiss Neurodevelopmental Outcome Registry for Children with CHD (ORCHID):

Cristina Borradori-Tolsa, Maya S. Bouhabib, Mark Brotzmann, Berenice Bubl, Seraina Calonder Faas, Robert Cesnjevar, Hitendu Dave, Margreet Duetz, Larina Eisenhut, Ruth Etter, Therese Fahrni, Katharina Fuhrer-Kradolfer, Martin Glöckler, Barbara Goeggel-Simonetti, Amir-Reza Hosseinpour, Damian Hutter, Alexander Kadner, Lena Kaiser, Christa Killer, Hannah Kümin, Rachel Kusche, Beatrice Latal, Marie Pascale Metrailler, Julia C. Natterer, Marc Raphael Pfluger, Angelo Polito, René Pretre, Regula Schmid, Juliane Schneider, Nicole Sekarski, Matthias Siepe, Tornike Sologashvili, Ursula Speckle, Alexandra Stöckli, Letizia von Laer, David Wille, Catherina Wolf
